# Registration-Based Organ Positioning and Joint Segmentation Method for Liver and Tumor Segmentation

**DOI:** 10.1155/2018/8536854

**Published:** 2018-09-24

**Authors:** Huiyan Jiang, Shaojie Li, Siqi Li

**Affiliations:** ^1^Department of Software College, Northeastern University, Shenyang 110819, China; ^2^Department of Sino-Dutch Biomedical and Information Engineering School, Northeastern University, Shenyang 110819, China

## Abstract

The automated segmentation of liver and tumor from CT images is of great importance in medical diagnoses and clinical treatment. However, accurate and automatic segmentation of liver and tumor is generally complicated due to the complex anatomical structures and low contrast. This paper proposes a registration-based organ positioning (ROP) and joint segmentation method for liver and tumor segmentation from CT images. First, a ROP method is developed to obtain liver's bounding box accurately and efficiently. Second, a joint segmentation method based on fuzzy c-means (FCM) and extreme learning machine (ELM) is designed to perform coarse liver segmentation. Third, the coarse segmentation is regarded as the initial contour of active contour model (ACM) to refine liver boundary by considering the topological information. Finally, tumor segmentation is performed using another ELM. Experiments on two datasets demonstrate the performance advantages of our proposed method compared with other related works.

## 1. Introduction

Liver cancer is one of the most commonly cancers with high mortality and poor prognosis all over the world [1]. According to the statistics, about 782,000 new cases were diagnosed with liver cancer and about 745,000 patients died of this disease worldwide in 2012 [2]. Consequently, accurate detection and segmentation of liver and tumor from computed tomography (CT) images are of great significance in clinical treatments. CT has been widely applied to noninvasive diagnosis of hepatic disease due to the latest advances in medical imaging technology [3]. Generally, experienced radiologists often delineate liver boundary manually slice by slice which is time-consuming and operator-subjective [4]. Thus, some semiautomatic and automatic segmentation methods have been proposed and applied to clinical applications. Many researchers have provided public databases and conducted liver segmentation competitions to facilitate the research of accurate segmentation algorithms [5].

Recently, numerous liver and tumor segmentation methods have been proposed [5, 6]. Ruskó et al. [7] proposed an adaptive region growing method for liver segmentation, in which liver is segmented by determined seed points and improved region growing criteria, and a postprocessing is then applied to refine liver shape. In [8], the original image is first segmented by density peak clustering. Then, the graph cuts are used to segment the liver in each slice. Finally, a vessel compensation method based on the border marching is used to increase the segmentation accuracy. This method may cause oversegmentation due to intensity overlap. In [9], a support vector machine (SVM) is used to achieve liver segmentation. First, features of image are extracted, and liver is then segmented by SVM classifier. Finally, the segmentation result is optimized by morphological operations. In this method, different features influence segmentation results a lot. Freiman et al. [10] used Bayesian classifier and morphological operations to achieve initial liver segmentation, whose boundary is used as initial contour of active contour model to segment liver accurately. Peng et al. [11] proposed a variational energy based method to delineate ambiguous liver edges from complex backgrounds. This method needs to initialize liver region manually, which limits clinical application. In [12], a novel superpixel and boundary sensitive convolutional neural network (SBBS-CNN) based method is used for automatic liver segmentation. CT images are segmented into superpixels which are then labeled by three classes: interior liver, liver boundary, and background. Finally, a deep CNN is built and trained to predict the probability of liver boundary. This method requires a large amount of training data and high experimental platform. Zeng et al. [13] presented modified graph cuts and feature detection based on vessel anatomic structure used for liver and liver vessel segmentation, respectively. In [14], Laplacian mesh optimization is used for liver segmentation from CT and MR images. An approximate 3D model of liver is initialized by a few manually generated contours, firstly. Then, the model is automatically deformed by Laplacian mesh optimization until it delineated liver accurately. This method requires liver shape initialization, which affects liver segmentation accuracy. Huang et al. [15] developed a fast extreme learning machine (ELM) based method for liver tumor detection and segmentation. Qian et al. [16] proposed an algorithm for medical image segmentation based on FCM and level set, in which initial contour of object is obtained through FCM and accurate segmentation is achieved by level set evolution. Dong et al. [17] presented a template matching framework based on an iterative probabilistic atlas for liver and spleen segmentation. First, a bounding box for candidate organ, which referred to the statistical geometric location, is detected. Then, the probabilistic atlas is regarded as a template to search the organ in corresponding bounding box by template matching technology. The results show that organ positioning can improve accuracy for multiorgan segmentation.

For uneven intensity of liver and tumor and low contrast between liver and other organs, accurate and automatic segmentation of liver and tumor from CT image is a challenging task. In this paper, we propose a registration-based organ positioning (ROP) and joint segmentation method for liver and tumor segmentation in a coarse-to-fine strategy. The main contributions of this paper are as follows. First, for useless information contained in CT images affecting the accuracy of liver segmentation, we first develop a ROP method which could find each liver's bounding box efficiently without inverse transformation by using undetermined and standard liver images as reference and float images, respectively, during registration. Second, to deal with undersegmentation caused by pixel-wise classification methods (such as FCM and ELM), a weighted fusion algorithm is proposed, in which segmentation results of FCM and ELM are fused by adaptively determined weights. Experiments demonstrate that our proposed method can achieve satisfactory segmentation.

The rest of this paper is organized as follows. In Section 2, the novel ROP and joint segmentation method is described in detail.  Section 3 presents experimental results and comparisons. Discussions and conclusions are summarized in Sections 4 and 5.

## 2. Methods

**Figure 1:**
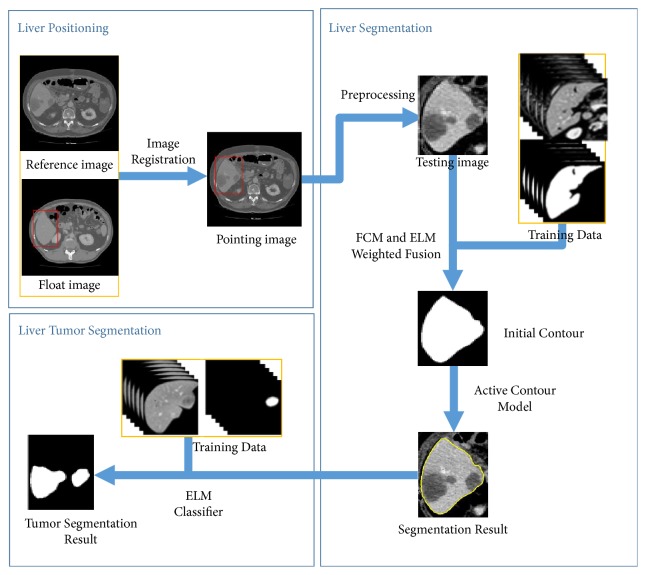
The flowchart of the proposed segmentation method for liver and liver tumor.

Figure 1 shows overview of the proposed segmentation method for liver and liver tumor, which includes three major steps: liver positioning based on ROP, liver segmentation by joint segmentation method in a coarse-to-fine strategy, and tumor segmentation using ELM classifier.

### 2.1. Registration-Based Organ Positioning

**Figure 2:**
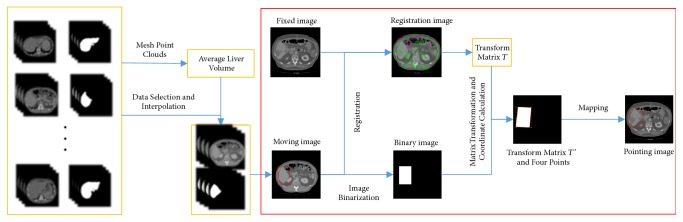
The process of the ROP method.

Generally, region of interest (ROI) of an organ, a three-dimensional bounding box as small as possible, is delineated using bounding box method which could contain the whole organ [25]. Specifically, for liver, the slice with the largest liver region is selected to determine its bounding box which is considered as liver bounding boxes of all slices [26]. However, there are two obvious shortages of the above methods. One is that it may introduce much unnecessary information which affects accuracy of segmentation results. The other one is its poor generation ability due to the physical differences between different patients (e.g., height, weight, and organ size). To address these two issues, this paper proposes a novel registration-based organ positioning (ROP) method for liver positioning. As shown in Figure 2, the proposed ROP method is mainly divided into the following three steps.

**Step 1 .**

The volume of each patient's liver is calculated according to all abdominal CT images from Chinese PLA General Hospital, firstly. Then, following the image size, the three-dimensional coordinate of each voxel is determined and the smallest liver region close to lungs is z = 1.60 approximately. Next, the mesh point cloud is used to create a set of three-dimensional points which could generate tetrahedral meshes, and the volume of surface mesh is calculated using the convex hull function. Finally, the average liver volume closest to the three-dimensional CT slices and the corresponding liver labels are selected via the calculated volume above.

**Step 2 .**

Following Step 1, the selected three-dimensional CT slices and the corresponding liver labels are interpolated along the Z-axis direction. The liver bounding box in corresponding CT slice is delineated using the liver label. Note that the above operation is performed on each CT slice after interpolation. The obtained liver bounding box in three-dimensional CT slice can be used as standard CT for image registration.

**Step 3 .**

Each CT image of the undetermined liver bounding box is taken as the reference image, and the standard CT image is used as the float image. Our intention of this step is registering float image to reference image. First, the optimizer and metrics used for coarse registration could be captured by cross-correlation method. Then, to improve registration accuracy, steps of optimizer and number of iterations are changed to get precise transform matrix *T*. The pixel in bounding box region and float image are labeled as 1 and 0, respectively. Then a new matrix *T*′ is obtained by the transform matrix *T*. Next, *T*′ is located in the two-dimensional coordinate system with the upper left corner as origin coordinates. Following the coordinates (X, Y) = (*x*_1_, *y*_1_), (*x*_2_, *y*_2_),…, (*x*_*n*_, *y*_*n*_) of all pixels in the matrix with label of 1, (*x*_*min*_, *y*_*min*_), (*x*_*min*_, *y*_*max*_), (*x*_*max*_, *y*_*max*_), and (*x*_*max*_, *y*_*min*_) are calculated by the minimum and maximum values of X and Y. Finally, a bounding box of liver in reference image is obtained through the four coordinate points.

The proposed ROP method has two important characteristics. First, different from other registration-based methods, the standard image is regarded as the float image, which could be used to obtain the different bounding boxes of different CT images with liver unlocated. It reduces the impacts produced by physical differences effectively. Second, the ROP method could improve the computational efficiency of finding bounding box noticeably without inverse registration.

### 2.2. Liver Segmentation

**Figure 3:**
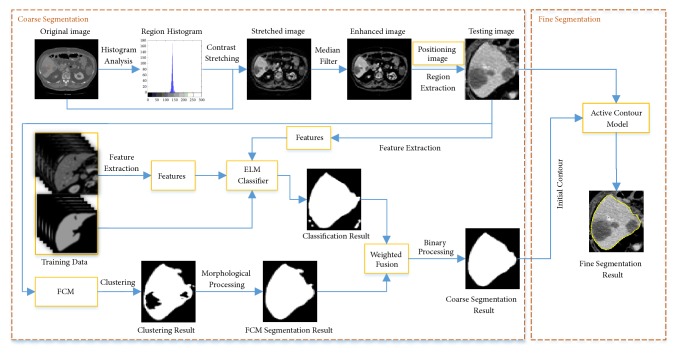
The process of liver segmentation.

As Figure 1 shows, the core of the liver segmentation method corresponds to object contour evolution in a coarse-to-fine strategy. The specific flowchart is shown in Figure 3. The coarse segmentation is performed in each bounding box by jointing FCM with ELM, and then the initially segmented liver boundary is refined using ACM. From the experimental results, the proposed joint segmentation method could tackle the low contrast and blurry boundary between liver and neighbor organs effectively. The following sections describe the proposed liver segmentation method specifically.

#### 2.2.1. Coarse Segmentation

**Figure 4:**
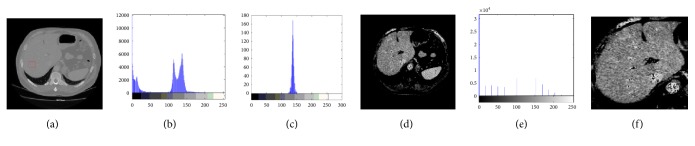
Image preprocessing. (a) An original image. (b) The histogram of (a). (c) The histogram of the red rectangular region. (d) The contrast enhanced image. (e) The histogram of (d). (f) The liver's bounding box on the enhanced image.

*(a) Preprocessing. *For more accurate segmentation, preprocessing of CT images is an important prerequisite to improve quality and contrast. The concrete steps are as follows. First, a small region of each liver is randomly extracted on abdominal CT image. Then, the gray range of the liver is determined by statistically analyzing the gray histogram of the extracted image patch which is commonly from 130 to 150. Next, pixels with gray value which corresponds to the peak of histogram are preserved, while other pixels' intensities are set to 0. The liver peak is balanced using the contrast stretching method [27]. Finally, a median filter (5×5) is utilized to smooth the image. Following the image preprocessing, each liver's bounding box on the enhanced images is obtained with ROP method. An example illustrating the image preprocessing method is shown in Figure 4.  Figure 4(a) is an original CT image and the red rectangle is the selected liver region.  Figure 4(b) is the gray histogram of Figure 4(a), and Figure 4(c) represents the gray histogram of the red rectangle region.  Figure 4(d) is the contrast enhanced image, and Figure 4(e) presents the gray histogram of Figure 4(d).  Figure 4(f) is the obtained liver's bounding box region based on ROP.

*(b) FCM-Based Segmentation. *Typically, fuzzy c-means (FCM) clustering algorithm, an improvement of hard k-means algorithm [9], has been proposed to optimize the objective function for the classification of the dataset iterative methods. It classifies each data point based on the similarity between the data point and all predefined classes, and the data point is classified into the particular class with the highest similarity. In addition, the FCM is an iterative clustering algorithm to classify samples by minimizing the cost function. The specific processes of FCM-based segmentation are as follows. First, since each liver's bounding box region contains liver, fat, background, and other organs, the number of clusters is set to 4. Second, the biggest connectivity component of clustering results is considered as candidate liver region. Finally, the FCM-based segmentation result is obtained by performing morphological operations on candidate segmented liver.

*(c) ELM-Based Segmentation. *The brief theory of ELM has been described in [28]. ELM is applied in many tasks, such as image segmentation, classification, and recognition [28, 29]. It provides good performance at a very fast learning speed. Concretely, ELM algorithm works as follows. Given a training set *N*, a single-hidden layer feedforward network (SLFN) with *L* hidden nodes can encode the training data using random initial weight *w*_*i*_ and bias *b*_*i*_. Following this process, we can obtain the hidden layer output matrix of SLFN (*H*). SLFN can approximate these *N* data with zero error. In other words, training SLFN is equivalent to finding a least-squares solution *β* = *H*^†^*T*, where *β* and *T* represent the output weight and the truth label, respectively. *H*^†^ denotes the Moore-Penrose generalized inverse of *H*.

Generally, 2D and 3D image segmentation using ELM, which are both considered as classification of objects and backgrounds, are based on using pixel, path, or surface as element. Each element's feature vector is utilized as input of ELM. In our experiment, we set the number of hidden layer neurons and the activation function as 630 and sin(*w*_*i*_*x*_*i*_ + *b*_*i*_), respectively [30]. We extract the features of each pixel inside liver's bounding box region (Figure 4(f)) to construct the feature vectors. As features used in ELM will influence segmentation result, appropriate feature extraction is important. In this paper, several feature extraction methods, including gray-intensity, the first order derivative of 2D Gaussian filtering, the second order derivative of Gaussian filtering in x and y direction, local standard deviation, bottom-hat filtering, phase consistency, canny edge, Harris, local binary patterns (LBP) [31], Gabor filter, Hessian, neighborhood mean and variance, entropy, intensity cooccurrence, Law's texture [32], and sum and difference histogram [33], are adopted to capture the local features of each pixel. To segment liver from background, we label the features extracted on the liver's bounding box region contour as 1 and 0, respectively. We integrate all the above features into the eigenvector of each pixel to train an ELM classifier. We train the ELM-based segmentation model using 10-fold cross validation. Considering 20 patients' CT scans, each cross validation is performed with different patient combinations (18 cases for training and the rest for testing). After the 10-fold cross validation, an optimal ELM model is obtained for automatic liver detection and segmentation. Finally, the segmentation result is optimized by filtering and binary processing.

*(d) Weighted Fusion of FCM and ELM. *According to experimental analysis, FCM-based segmentation often leads to oversegmentation. On the contrary, ELM-based segmentation generally causes undersegmentation. To address these issues, we propose a weighted fusion method based on mutual information of FCM-based and ELM-based segmentation results. The weights are determined automatically according to the segmentation accuracies of training data. Specifically, the accuracies of the FCM-based and ELM-based segmentation results on training slice *i* are *A*_*F*_^*i*^ and *A*_*E*_^*i*^, and the weights of training slice *i* are *A*_*F*_^*i*^/(*A*_*F*_^*i*^ + *A*_*E*_^*i*^) and *A*_*E*_^*i*^/(*A*_*F*_^*i*^ + *A*_*E*_^*i*^), respectively. Finally, the optimal weight set is obtained by calculating the mean value of the largest (*S*_*ω*_^*i*^(*i*=1,2,…,100)). The coarse segmented liver which is used for fine segmentation is obtained by implementing median filtering and binary processing on the fused result.

#### 2.2.2. Fine Segmentation

FCM- and ELM-based segmentation methods are both performed based on the pixel-wise classification and their own efficiency superiority. These two methods could not refine liver's boundary based on topological information. Therefore, we employ a coarse-to-fine method using the active contour model (ACM) [34] to refine liver's boundary. The boundary of coarse segmented liver is used as initial contour for ACM. Then, the contour is optimized under the influence of external internal force, where the internal force is the energy term, which keeps the contour in continuity and smoothness during the evolution; meanwhile the external force is determined by the information of image, such as gradient of image, which drives the contour converge toward target [35]. Finally, the accurate liver boundary is acquired by considering the pixel-wise and topological information simultaneously.

### 2.3. Liver Tumor Segmentation

As previously mentioned, the task of tumor segmentation is accomplished with ELM classifier. Similar to ELM-based liver segmentation method (Section 2.2.1), the pixels inside liver should be classified as tumor and nontumor. The steps for tumor segmentation are as follows. First, based on the liver segmentation result, the same 126-dimension features of each pixel inside liver are extracted to construct feature matrix. Then, another ELM model used for segmenting tumor is trained by the new feature matrix. Note that the same 10-fold cross validation method is performed in training process to obtain the optimal ELM model.

## 3. Experiments

**Figure 5:**
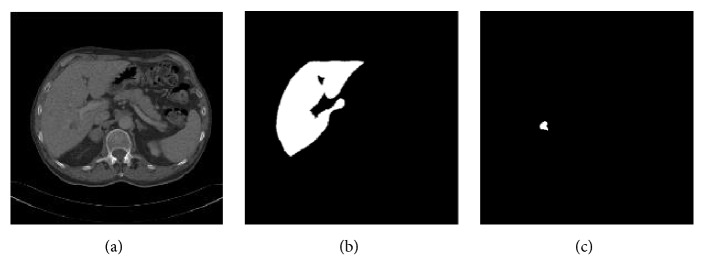
An example of the CT image and its labels (liver and tumor). (a) An abdominal CT image. (b) The liver label. (c) The tumor label.

Our experiments contain two databases: one is 3Dircadb database which involves 20 patients' CT data, and the other contains abdominal CT images of 120 patients obtained from the Department of Radiology, Chinese PLA General Hospital, Shenyang. For 3Dircadb database, the number of slices, pixel spacing, and interslice resolution varied from 40 to 159, 0.56 to 0.81mm, and 1.25 to 4.0mm, respectively. For local database, the number of slices in each volume varied from 70 to 135 with 512×512 resolution. Pixel spacing is 0.78mm and thickness between each slice is 1.60 mm. The liver and tumor are labeled by radiologists. All CT images of 120 patients from Chinese PLA General Hospital are utilized to develop a novel ROP method and the remaining CT images from 3Dircadb database are employed to train and test our proposed joint segmentation method. We evaluate the proposed segmentation method using all CT images from 3Dircadb database with 10-fold cross validation. An example of CT image and its labels (liver and tumor) from 3Dircadb database is shown in Figure 5.  Figure 5(a) shows an abdominal CT image, Figures 5(b) and 5(c) show the labels of liver and tumor, respectively. In addition, the experiment is implemented using MATLAB R2014a on Windows 7 OS based computer with Intel(R) Core (TM) i7-4790 CPU @ 3.6GHz, 16G RAM, 930G hard disk.

### 3.1. Evaluation Measures

To evaluate the performance of the proposed method quantitatively, six criteria are used for segmentation evaluation [15, 36] which are volumetric overlap error (VOE), relative volume difference (RVD), average symmetric distance (ASD), root mean square symmetric surface distance (RMSD), maximum symmetric surface distance (MSD), and Dice similarity coefficient (DSC). Specifically, the six evaluation criteria are described as follows. Note that the object in ground truth is *A*, and *B* is the predicted object. Volumetric overlap error (VOE) is the complement of the Jaccard coefficient.

(1) $$\begin{array}{c} VOE\left(\;A,B\;\right)=100\times \left(\;1-\frac{\left|A\cap B\right|}{\left|A\cup B\right|}\;\right) \end{array}$$

Relative Volume Difference (RVD) is an asymmetric which is defined as follows.

(2) $$\begin{array}{c} RVD\left(\;A,B\;\right)=100\times \frac{\left|B\right|-\left|A\right|}{\left|A\right|} \end{array}$$

Average symmetric distance (ASD): Let *S*(*A*) denote the set of surface voxels of *A*. The shortest distance of an arbitrary voxel *v* to *S*(*A*) is defined as follows.

(3) dv,SA=minsA∈SA⁡v−sA

(4) ASDA,B=1SA+SB∑sA∈SAdsA,SB+∑sB∈SBdsB,SA

Root mean square symmetric surface distance (RMSD) is defined as follows.

(5) RMSDA,B=1SA+SB×∑sA∈SAd2sA,SB+∑sB∈SBd2sB,SA

Maximum symmetric surface distance (MSD) is similar to ASD, which is defined as follows.

(6) MSDA,B=max⁡maxsA∈SA⁡dsA,SB,maxsB∈SB⁡dsB,SA

The Dice Score (DICE) is evaluated as follows.

(7) $$\begin{array}{c} DICE\left(\;A,B\;\right)=100\times \frac{2\left|A\cap B\right|}{\left|A\right|+\left|B\right|} \end{array}$$

### 3.2. Results

**Figure 6:**
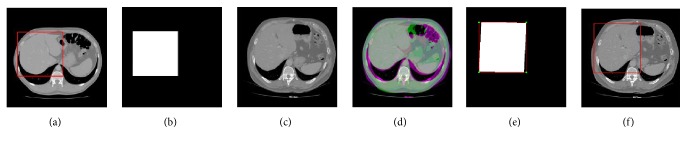
An example of liver positioning using ROP method. (a) A CT slice with the closest average liver volume and liver's bounding box region (red). (b) The binary image. (c) Another image from different patient. (d) The corresponding image registration result. (e) Four new vertexes (green) based on the transformation result. (f) Obtained liver positioning result.

**Figure 7:**
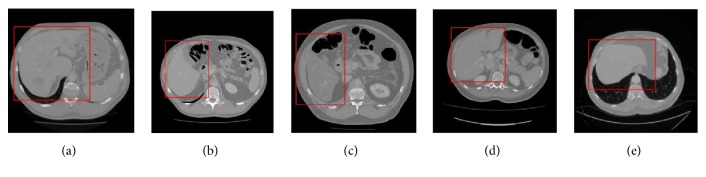
The liver positioning results of some other slices. (a)~(e) The liver positioning results with different liver shapes on different patient slices.

**Figure 8:**
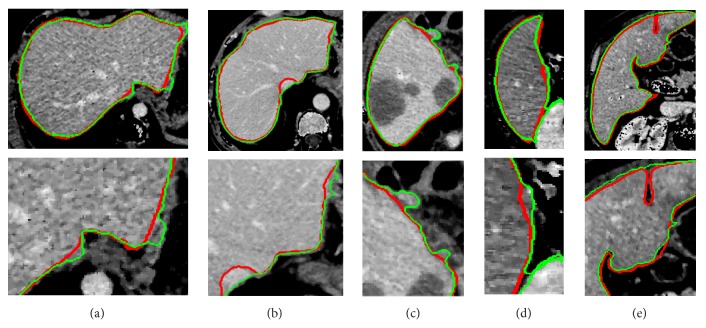
Comparisons between FCM-based segmentation results and ground truth. (a)~(e) The FCM-based segmentation results of five different slices. The first row is the comparisons between the segmentation results and ground truth, and the second row is the zoomed local areas corresponding to first row images (green is FCM-based segmentation result; red is ground truth).

**Figure 9:**
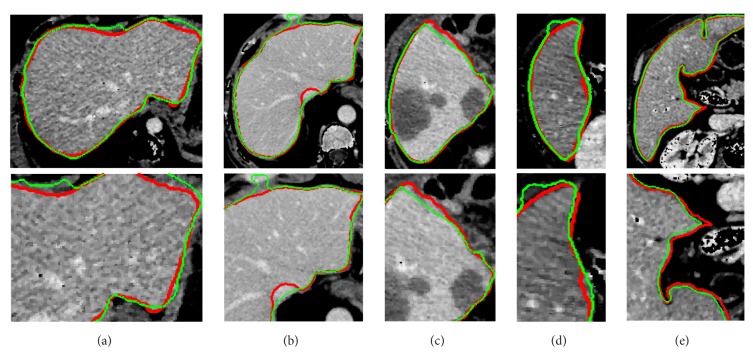
Comparisons between ELM-based segmentation results and ground truth. (a)~(e) The ELM-based segmentation results. The first row is the comparisons between segmentation results and ground truth, and the second row is the local areas corresponding to first row image (green is ELM segmentation result; red is ground truth).

**Figure 10:**
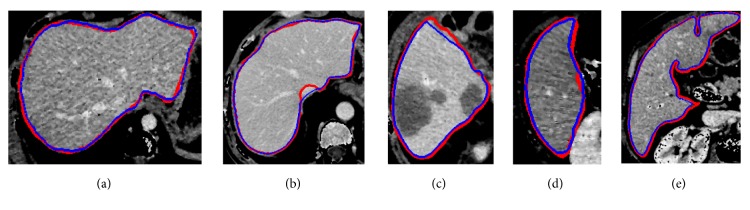
Comparisons between coarse segmentation results and ground truth. (a)~(e) The coarse segmentation results of five different slices (blue is coarse segmentation result; red is ground truth).

**Figure 11:**
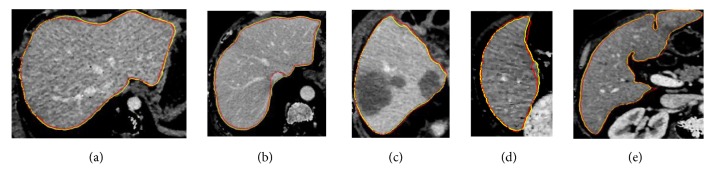
Comparisons between fine segmentation results and ground truth (yellow is fine segmentation result; red is ground truth).

**Figure 12:**
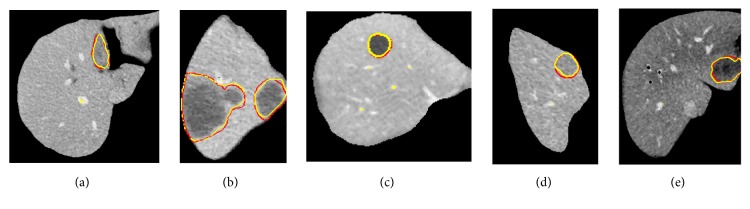
Comparisons between tumor segmentation results and ground truth (yellow is tumor segmentation result; red is ground truth).

To evaluate the effectiveness of the proposed method, we test the proposed segmentation model with 120 volumes from Chinese PLA General Hospital whose average liver volume is 1720 cm^3^.  Figure 6 presents an example of liver's bounding box region determination using ROP method.  Figure 6(a) shows a CT slice with the closest average liver volume and the red bounding box contains complete liver.  Figure 6(b) is the binary image.  Figure 6(c) is a CT image of another patient.  Figure 6(d) shows the corresponding registration result.  Figure 6(e) is the result of transformation relative to Figure 6(b). Note that four green points, (*x*_*min*_, *y*_*min*_), (*x*_*min*_, *y*_*max*_), (*x*_*max*_, *y*_*max*_), and (*x*_*max*_, *y*_*min*_), are new vertexes.  Figure 6(f) shows the result of the liver positioning.  Figure 7 shows liver positioning results of different slices. It can be observed that ROP method could locate liver accurately and the obtained bounding box contains complete boundary information.  Figure 8 presents the FCM-based segmentation results of 5 CT slices. The upper row shows the comparisons between FCM-based results and ground truth, and the lower row shows the local patches corresponding to the upper row images. Green and red curves are FCM-based segmentation results and ground truth, respectively. We observe that there are some around liver.  Figure 9 shows the ELM-based segmentation results of the same 5 CT slices. The first row shows the comparisons between ELM-based results and ground truth, and the second row exhibits the local patches of corresponding image from the first row. It is observed that the ELM-based segmentation method is more likely to cause undersegmentation (Figure 9(c)).  Figure 10 shows the coarse segmentation results fused with FCM- and ELM-based segmentation results.  Figure 11 shows the refined segmentation results of 5 slices using ACM. By considering the topological information, the liver boundaries are closer to ground truth. Finally, Figure 12 shows tumor segmentation results of other 5 slices. The comparisons with manually segmented tumor demonstrate the proposed ELM-based segmentation with high accuracy.

### 3.3. Performance Comparisons

Comparison results between our proposed method and other methods are presented in this section. The results of each metric are calculated as the mean values on the whole database.  Table 1 presents the different evaluation indexes to verify the effectiveness of our proposed liver segmentation method. It can be seen that the proposed method performs best in terms of VOE, RMSD, MSD, and DSC. It performs slight inferior to [20] in ASD. For the contrastive methods, Wimmer et al. [18] used liver detection and probabilistic active shape model to execute liver segmentation. Linguraru et al. [19] combined normalized probabilistic atlases and enhanced estimates to segment liver. Dong et al. [17] employed the liver location and probabilistic atlas to conduct liver segmentation. Finally, Hu et al. [20] utilized a convolutional neural network (CNN) to predict the prior information for liver segmentation.  Table 1 also shows the running time for testing. The computation time of our method is about 89s per CT volume, which is the lowest among these methods.  Table 2 shows the evaluation indexes for tumor segmentation of our proposed method and other contrastive methods. Li et al. [21] proposed that a likelihood energy approach is better. Christ et al. [22] employed cascaded fully convolutional neural networks (CFCNs) and dense 3D conditional random fields (CRFs) to segment liver and lesions automatically. Kumar et al. [23] proposed an alternative fuzzy c-means (AFCM) for tumor segmentation. Zhou et al. [24] used an SVM-based classifier to classify tumor voxels. We can see that the ELM-based segmentation method achieves the Dice score of 94.8% for tumor segmentation. In addition, the proposed method achieves 13.5% VOE, -4.7% RVD, 1.4 mm ASD, and 23.2 mm MSD. The comparative results show that the proposed method is superior to the other four methods with regard to the Dice score, ASD, and MSD.

## 4. Discussions

Different from other methods of determining the bounding box, the proposed ROP method regards images with uncertain liver location as reference images and considers an image with the certain liver location as float image. Then, the liver's bounding box is determined by image registration. As Figure 7 shows, the ROP method can obtain bounding boxes of livers with different shapes accurately. Noticeably, ROP improves the computational efficiency due to no inverse registration.

For liver segmentation, FCM- and ELM-based segmentation methods both cause some incorrect segmentations (oversegmentation or undersegmentation); the weight fused method could address these issues and generate coarse segmentation result, which is regarded as initial contour for ACM. It is important to obtain a relatively accurate coarse segmentation result, as ACM is sensitive to initial contour.  Figure 11 demonstrates that ACM considering topological information could compensate for the deficiency of FCM- and ELM-based methods. In addition, FCM and ELM possess better extensibility and faster learning rate, and the proposed coarse segmentation method thus has obvious advantages in terms of segmentation time. Finally, following ELM-based segmentation method, the tumors are located and segmented successfully inside livers. The comparing results in Figure 12 show the superior performance of ELM-based method.

## 5. Conclusions

This paper proposes a novel registration-based organ positioning (ROP) and joint segmentation method for liver and tumor segmentation in a coarse-to-fine strategy. First, we develop a ROP method to locate liver and determine liver's bounding box based on image registration. Then, the weighted fused segmentation result is obtained with results of FCM- and ELM-based methods, which is regarded as the initial contour which is used for fine segmentation based on ACM. Finally, the tumor segmentation is performed using another ELM classifier. Experiment results demonstrate that our proposed ROP and joint segmentation method has higher positioning accuracy and better segmentation performance.

## Figures and Tables

**Table 1:** The evaluation indexes for liver segmentation between our proposed method and other contrastive methods.

Metric methods	VOE (%)	RVD(%)	ASD (mm)	RMSD (mm)	MSD (mm)	DSC (%)	Time (–)
[18]	6.47	1.04	1.02	2.00	18.32	96.65	3min
[19]	6.37	2.26	1.00	1.92	20.75	96.70	n/a
[17]	6.44	**0.01**	0.98	1.87	18.14	96.67	143s
[20]	5.35	-0.17	**0.84**	1.78	19.58	97.25	135s
Ours	**4.74**	1.44	0.89	**1.57**	**17.15**	**97.43**	**89s**

**Table 2:** The evaluation indexes for tumor segmentation between our proposed method and other contrastive methods.

Metric methods	VOE(%)	RVD(%)	ASD (mm)	MSD (mm)	DSC(%)
[21]	9.2	-11.2	1.6	28.2	*∗*
[22]	10.7	-1.4	1.5	24.0	94.3
[23]	*∗*	*∗*	*∗*	*∗*	91.7
[24]	25.7	17.9	1.6	*∗*	87.2
Ours	13.5	-4.7	1.4	23.2	**94.8**

## Data Availability

The data used to support the findings of this study are available from the corresponding author upon request.
